# Comparative Age-Period-Cohort Analysis Reveals Divergent Temporal Trajectories of Breast and Colorectal Cancer Among Women in Singapore

**DOI:** 10.3390/cancers18213538

**Published:** 2026-11-03

**Authors:** Josephine Yi Hui Pan, Mengyu Li, Dawn Qingqing Chong, Sunny Hei Wong, Iain Bee Huat Tan, Xueling Sim, Kee Seng Chia, Jingmei Li

**Affiliations:** 1 Genome Institute of Singapore (GIS), Agency for Science, Technology and Research (A*STAR), 60 Biopolis St, Genome, #02-01, Singapore 138672, Singapore; 2 Saw Swee Hock School of Public Health, National University of Singapore and National University Health System, Singapore 117549, Singapore; 3 National Cancer Centre Singapore (NCCS), Singapore Health Services ( SingHealth), Singapore 168583, Singapore; 4 Duke-National University of Singapore Medical School, Singapore 169857, Singapore; 5 Lee Kong Chian School of Medicine, Nanyang Technological University, Singapore 308232, Singapore; 6 Department of Gastroenterology and Hepatology, Tan Tock Seng Hospital, Singapore 308433, Singapore; 7 Department of Surgery, Yong Loo Lin School of Medicine, National University of Singapore and National University Health System, Singapore 119228, Singapore

**Keywords:** age–period–cohort analysis, breast cancer, colorectal cancer, cancer incidence trends, ethnic disparities, generational cohort effects

## Abstract

**Background**: Breast cancer (BC) and colorectal cancer (CRC) are leading malignancies among women in Singapore, but their temporal drivers may differ despite occurring within the same healthcare system. We compared both cancers using a common analytical framework across major ethnic groups to identify ethnic differences that may be hidden in national trends. **Objective:** To disentangle age, period, and cohort effects on incidence trends among women aged 25–84 years by ethnicity (Chinese, Malay, Indian). **Methods:** We analysed population-based Singapore Cancer Registry data from 1968 to 2022. We calculated age-standardised incidence rates using the Segi world standard population and fitted age–period–cohort models separately by cancer and ethnicity. Nested models were compared using quasi-likelihood F tests with Benjamini–Hochberg-adjusted *p*-values. Overdispersion was assessed using Pearson χ^2^/df. For BC, quasi-Poisson regression models, which extend Poisson regression by allowing the variance to exceed the mean, tested whether cohort associations differed between women aged <49 and ≥49 years (age-band midpoint <49 versus ≥49). Sensitivity analyses examined the influence of imputing suppressed counts in sparse strata. **Results:** We analysed 53,514 BC and 25,658 CRC cases. BC incidence increased across ethnicities, mostly along a linear drift that cannot be attributed to period or cohort. Beyond this drift, non-linear cohort effects contributed more to model fit than non-linear period effects. Cohort curvature rose up to the Baby Boomer cohorts and fell thereafter, indicating a slower increase than the linear trend in later cohorts rather than a decline in absolute risk. The age-group by birth-cohort interaction significantly improved model fit overall and among Chinese women (*p* = 0.00339), but not among Indian (*p* = 0.0873) or Malay women (*p* = 0.672). Among Indian women, the age–period model had the lowest AIC, but nested-model comparisons depended on the value imputed for suppressed counts, so the relative contributions of period and cohort effects could not be determined reliably. Cohort effects were more strongly supported among Malay women, whereas both period and cohort effects contributed to model fit among Chinese women. Early incidence trends and nested-model comparisons among Indian women were sensitive to suppressed-count imputation. **Conclusions:** Beyond the linear drift, BC trends are explained more strongly by cohort curvature than by period curvature. This is consistent with a role for generational changes in risk exposures, which this study did not measure directly. CRC showed ethnicity-specific period and cohort associations, although their relative contributions among Indian women remained uncertain because of sensitivity to suppressed-count imputation.

## Simple Summary

This study compares 55-year trends in breast cancer (BC) and colorectal cancer (CRC) among Singaporean women, and whether they differ by ethnicity. Using national cancer-registry data, we examined how cancer incidence varied with age, calendar period and birth cohort, while accounting for the difficulty of separating their overlapping trends. BC trends were more strongly associated with cohort effects. Beyond the steady overall increase, the generational component rose up to the Baby Boomers. In later generations, incidence continued to rise but more slowly than the overall trend. This may relate to later childbearing, fewer births and lifestyle changes, but this study could not test these explanations. CRC trend drivers vary by ethnicity. For Indian women, age and calendar year appeared to describe the pattern of CRC rates, but this result changed when we altered how very small counts were handled, so it should be read cautiously. For Malay women, being born in a particular generation was a stronger explanation for the pattern. For Chinese women, both calendar-year changes and generational differences helped explain the trends. Unlike earlier Singapore studies that examined each cancer separately, we compare both in women using the same methods and report results by generation (Silent Generation, Baby Boomers, Gen X, Millennials) to better link findings to historical and policy changes. The findings suggest that generational exposures may deserve attention in BC prevention planning and that CRC strategies may need to consider ethnic differences.

## 1. Introduction

Cancer represents a major health challenge in a rapidly ageing population [1]. With one in four Singaporean residents expected to be diagnosed by age 75 years [2], the disease imposes a significant clinical and financial burden on households and public healthcare spending [3]. Understanding population cancer trends and risk factors is therefore integral for informing public policy, resource allocation and prevention strategies.

Breast cancer (BC) is the most commonly diagnosed cancer among women in Singapore (29.9% of all cancers in women), followed by colorectal cancer (CRC) (12.6%), according to the Singapore Cancer Registry Annual Report 2023 [2]. BC is predominantly associated with cumulative hormonal and reproductive exposures [4], whereas CRC is more strongly associated with metabolic, dietary and gut microbiome-related factors [5]. Both have undergone marked changes in incidence over recent decades. BC incidence has increased steadily across rapidly industrialising Asian societies, whereas female CRC has followed more heterogeneous temporal patterns, increasing in emerging economies but plateauing or declining in more established healthcare systems [6,7]. Comparing BC and CRC within the same female population provides an opportunity to examine how common societal changes relate to cancers with distinct biological mechanisms.

Temporal changes in cancer incidence reflect the combined influence of biological ageing, secular changes affecting the entire population, and generational differences in cumulative risk exposures. Age–period–cohort (APC) analysis provides an epidemiological framework for characterising age-related incidence patterns and identifiable nonlinear period and cohort variation, while accounting for the non-identifiability of their linear components [8]. Age effects reflect age-related physiological susceptibility and the accumulation of carcinogenic exposures. Period effects capture influences affecting all age groups, including organised screening programmes, advances in diagnostic technology and changes in disease classification. Cohort effects represent generational differences associated with early-life, reproductive, lifestyle and environmental exposures [9].

Singapore provides a unique setting for APC analysis because of the rapid pace of its economic and epidemiological transition in the past six decades and its multi-ethnic population [10,11]. This allows an evaluation of whether these societal changes influenced ethnic groups similarly. During this transition, the country transformed from a developing trading port into a high-income, urbanised nation. Alongside this, reproductive behaviour and lifestyle changed. Fertility declined, childbearing was delayed, nulliparity increased, breastfeeding duration shortened, diets shifted toward Westernised patterns, metabolic disease rose, and workforce participation grew [12,13].

Singapore also established universal healthcare coverage, introduced organised BC and CRC screening programmes, and continued to improve diagnostic services [14,15,16]. Nevertheless, a common healthcare system does not guarantee equivalent access to or utilisation of preventive and diagnostic services across ethnic groups. Ethnic differences have been reported in screening participation, health-seeking behaviour and barriers to cancer care, as well as differences in cultural practices, dietary habits, reproductive characteristics and metabolic risk profiles [17,18,19,20]. Ethnic differences in incidence may thus reflect two broad factors. The first is cumulative risk-factor profiles. The second is differences in healthcare access, screening uptake, diagnostic pathways and disease ascertainment. Both may vary over time. We therefore performed ethnically stratified temporal-trend analysis.

Previous APC studies in Singapore have characterised temporal trends in BC and CRC separately [21,22,23,24]. A recent analysis of Singapore cancer cases diagnosed between 2000 and 2021 found that period and cohort effects remained evident [25]. Generations that experienced the most consequential shifts in lifestyle and fertility are now approaching higher-risk ages for BC and CRC, and Singapore’s screening initiatives have matured. We therefore sought to determine whether cohort-associated risks have stabilised or continued to evolve across cancers and ethnic groups. In this nationwide, population-based study spanning 55 years (1968–2022), we applied a common analytical framework to comprehensive cancer registry data to (i) compare age, period and cohort dynamics of BC and CRC among Chinese, Malay and Indian women and (ii) interpret these patterns in light of documented reproductive, metabolic and healthcare transitions in Singapore. We aim to make a formal comparison of the relative contributions of period versus cohort processes for both BC and CRC, building on prior APC work and emphasising ethnic heterogeneity that is obscured in aggregated national trends.

## 2. Methods

### 2.1. Data Source and Study Population

This nationwide population-based ecological study examined temporal trends in BC and CRC incidence among women aged 25–84 years in Singapore between 1968 and 2022 using data from the Singapore Cancer Registry, a mandatory, population-wide registry with near-complete ascertainment of cancer diagnoses across the country under the National Registry of Diseases Office (NRDO) [26].

Cancer incidence counts and population denominators were obtained. The cancer incidence dataset included counts by (i) age at diagnosis (5-year intervals), (ii) sex, (iii) ethnicity, (iv) year of diagnosis (5-year calendar intervals), and (v) cancer site (i.e., breast or colon and rectum). Between 1968 and 2022, the Singapore Cancer Registry recorded an estimated 56,104 BC cases and 28,760 CRC cases among women. Cell-suppression values (<5 incident cases) in the registry were assigned the value of 3 to preserve sparse strata. The population dataset included denominators by (i) age (5-year intervals), (ii) sex, (iii) ethnicity, and (iv) calendar year. For each cancer site and ethnic group included in the analyses, a complete age–period grid was constructed, with person-years available for every cell. Age–period cells absent from the case extract were assigned zero cases and retained with their corresponding person-years. These zero-filled cells were distinguished from cells explicitly reported as “<5”, which were assigned a count of 3 in the main analysis.

Ethnicity was categorised using Singapore’s Chinese, Malay, Indian and Others (CMIO) framework, which is used in national population statistics [27]. Ethnicity in the cancer registry was based on recorded race information, with cases linked by the registry to national registration records using unique personal identifiers [28].

Following the introduction of double-barrelled race registration in 2011, the first-listed race was used for statistical classification [29]. Individuals not classified as Chinese, Malay or Indian were grouped under Others. Population denominators were obtained from national census and intercensal estimates using corresponding CMIO categories. Although we used these broad ethnic groupings throughout the analysis, changes in administrative recording practices, including the introduction of double-barrelled race registration, may have affected ethnic classification over time. The Others ethnicity category was excluded because of its heterogeneous composition, which limits the interpretation of ethnicity-specific incidence patterns.

Analyses were also restricted to women aged 25–84 years. Women younger than 25 years were excluded because of sparse cancer incidence counts, particularly in ethnicity-specific strata. Women aged 85 years and older were excluded to improve comparability across calendar periods and avoid potential instability associated with the open-ended oldest age category, whose population composition has changed with increasing longevity.

After excluding 1297 BC cases and 441 CRC cases from the Others ethnicity category, and a further 1293 BC cases and 2661 CRC cases among women younger than 25 years or aged 85 years and older, the final analytical dataset comprised 53,514 BC cases and 25,658 CRC cases. Analyses were performed separately for Chinese, Malay, and Indian women, as well as for these three ethnic groups combined.

This study was determined to meet the criteria for Review Not Required by the A*STAR Institutional Review Board (IRB Reference: 2026-061, 5 June 2026), consistent with its use of de-identified aggregate registry data supplied without individual-level identifiers.

### 2.2. Temporal and Demographic Parameterisation

The study period was structured into 5-year consecutive calendar intervals from 1968 to 1972 through 2018 to 2022 and represented by their respective midpoint years (e.g., 1970 for 1968–1972). Population denominators for each year were summed within each calendar interval for rate calculations to ensure consistency across all temporal blocks.

The age range was restricted to 25 to 84, comprising 12 five-year age groups with midpoints from 27 to 82 years. Cohort midpoints were obtained by subtracting each age-group midpoint from the corresponding period midyear.

### 2.3. Age-Standardised Rates (ASR) and Age-Specific Rates

ASR per 100,000 person-years were calculated for each unique ethnicity–cancer site–period combination using standard weights [30] derived from the Segi (1960) world standard population [31] for the 12 five-year age-brackets from age 25 to 84. Weights were normalised against the full 100,000-person standard population total, rather than within the restricted age range, so that the truncated (ages 25–84) ASR values are on the same scale across groups and periods but are lower than, and not directly comparable with, published all-age ASR. Gamma confidence intervals were calculated for ASR using the method described in [32]. ASR and their confidence intervals were smoothed using Loess smoothing (span = 0.75, degree = 2, weighted by incidence counts) and plotted against period midyears to show the underlying trend for each cancer separately. Age-specific incidence rates of BC and CRC in number of cases per 100,000 person-years were calculated with confidence intervals computed without weighting and similarly smoothed.

### 2.4. Statistical Analysis

APC models were fitted using Poisson regression with the logarithm of person-years included as an offset and age, period and cohort effects modelled using natural cubic splines [33] as implemented in the Epi package in R (v2.65) [34]. The default of 5 knots for each time scale, placed at the 10th, 30th, 50th, 70th and 90th percentiles of the case-weighted distributions of age, period and cohort (the outermost knots act as boundary knots, giving 4 df per effect and 3 df per non-linear component), was used. Year 1970 was used as the reference period and 1890 as the reference cohort. These references (the first period and approximately the earliest cohort) were chosen so that effects read as changes relative to the start of the observation window. The choice does not affect model fit, the net drift or the shape of the non-linear components. To overcome the identifiability problem, an orthogonal projection constraint was used to extract overall linear drift (Epi::apc.fit, parm = “AdCP”). Consequently, cohort and period rate ratios were detrended and represent (curvature) components of the cohort and period effects, respectively. The net linear drift is estimated separately and is not attributed to either [33]. Age effects were expressed as age-specific incidence rates in the reference cohort after period and cohort effects had been accounted for.

Sequential nested sub-models (age, age–drift, age–cohort, age–period–cohort, age–period) were compared to assess improvement of model fit with the addition of parameters. Model goodness-of-fit and residual overdispersion were quantified using the Pearson chi-square statistic divided by residual degrees of freedom (Pearson χ^2^/df) of the full APC model for each stratum, where values greater than 1 were taken to indicate overdispersion [35]. Deviance differences were evaluated with quasi-likelihood F tests [36], where deviance difference per degree of freedom was divided by the Pearson dispersion estimate from the full APC model for the corresponding cancer–ethnicity stratum. Dispersion factors were floored at 1 to prevent tests from becoming more liberal where underdispersion was detected. In the final analysis, no stratum had χ^2^/df below 1. Dispersion-corrected *p*-values were obtained from the F distribution (numerator df = test df; denominator df = residual df of the full APC model) and adjusted for multiple testing across ethnic and cancer strata using the Benjamini–Hochberg false discovery rate (FDR) procedure. Akaike Information Criterion (AIC) and deviance values were reported for reference and comparison but should be interpreted with caution given that their magnitude is largely dependent on data granularity [33]. A sensitivity analysis assessed whether imputing suppressed counts as 1, 2, 3 or 4 altered nested model comparisons for Indian women, who had the smallest incidence counts and highest proportion of imputed values.

Age 49 years was selected as the threshold for age-stratified analyses, informed by the reported average age at menopause of 49 years [37] and established differences in BC aetiology, tumour characteristics and incidence according to menopausal status [22,38,39]. As individual-level menopausal status was unavailable, this threshold served as an approximate proxy rather than a direct classification of menopausal status. Quasi-Poisson generalised linear models were fitted to breast cancer counts to account for overdispersion. These models assume $\mathrm{Var}\left(Y_{i}|X_{i}\right)=\phi \mu_{i}$, where $Y_{i}$ denotes the observed count, $\mu_{i}=E\left(Y_{i}|X_{i}\right)$ is its conditional expected value, and ϕ is an estimated dispersion parameter; ϕ>1 indicates overdispersion. Models included natural cubic splines of birth cohort midyear and age midyear, each with 5 degrees of freedom, an age-group (classified according to whether the midpoint of the five-year age band was <49 or ≥49 by age-band midpoint: bands 25–29 to 45–49 versus 50–54 to 80–84, so that the boundary approximates the mean age at menopause) main effect, and an interaction between age group and the birth cohort spline. A log link and the logarithm of person-years were used as the offset. An F-test compared the interaction model with a nested model retaining all main effects but excluding the interaction to assess whether birth cohort–BC incidence associations differed between age groups (unadjusted for multiple comparisons).

Period and cohort effects were expressed as period rate ratio (P-RR) and cohort rate ratio (C-RR) against age and birth cohort midyear, respectively. Birth cohort effect plots were stratified using widely accepted generational definitions to avoid arbitrary cut-offs: the Silent Generation (born before 1946), Baby Boomers (born 1946–1964), Gen X (born 1965–1980), and Millennials (born 1981–1996). No cases were observed among Gen Z (born after 1996) [40]. Generational labels are used solely as descriptive shorthand for groups of adjacent birth cohorts. These boundaries were defined in Western contexts and may not correspond to Singapore’s demographic and historical experience. All inferences were therefore based on birth-cohort analysis rather than on the generational categories themselves. Age effect plots were further stratified by age group (<49 versus ≥49 years).

All analyses were carried out using R v4.6.0 (released 24 April 2026) in RStudio (v2026.07.1+147). The analysis code supporting the findings of this study is openly available in the Zenodo repository at https://doi.org/10.5281/zenodo.23726980. The repository contains the complete R code used for data cleaning, age-standardised and age-specific rate calculations, APC model fitting, sensitivity analyses, menopause-group stratified GLM analysis, and figure generation.

## 3. Results

### 3.1. Overall Temporal Trends in ASR

**Figure 1:**
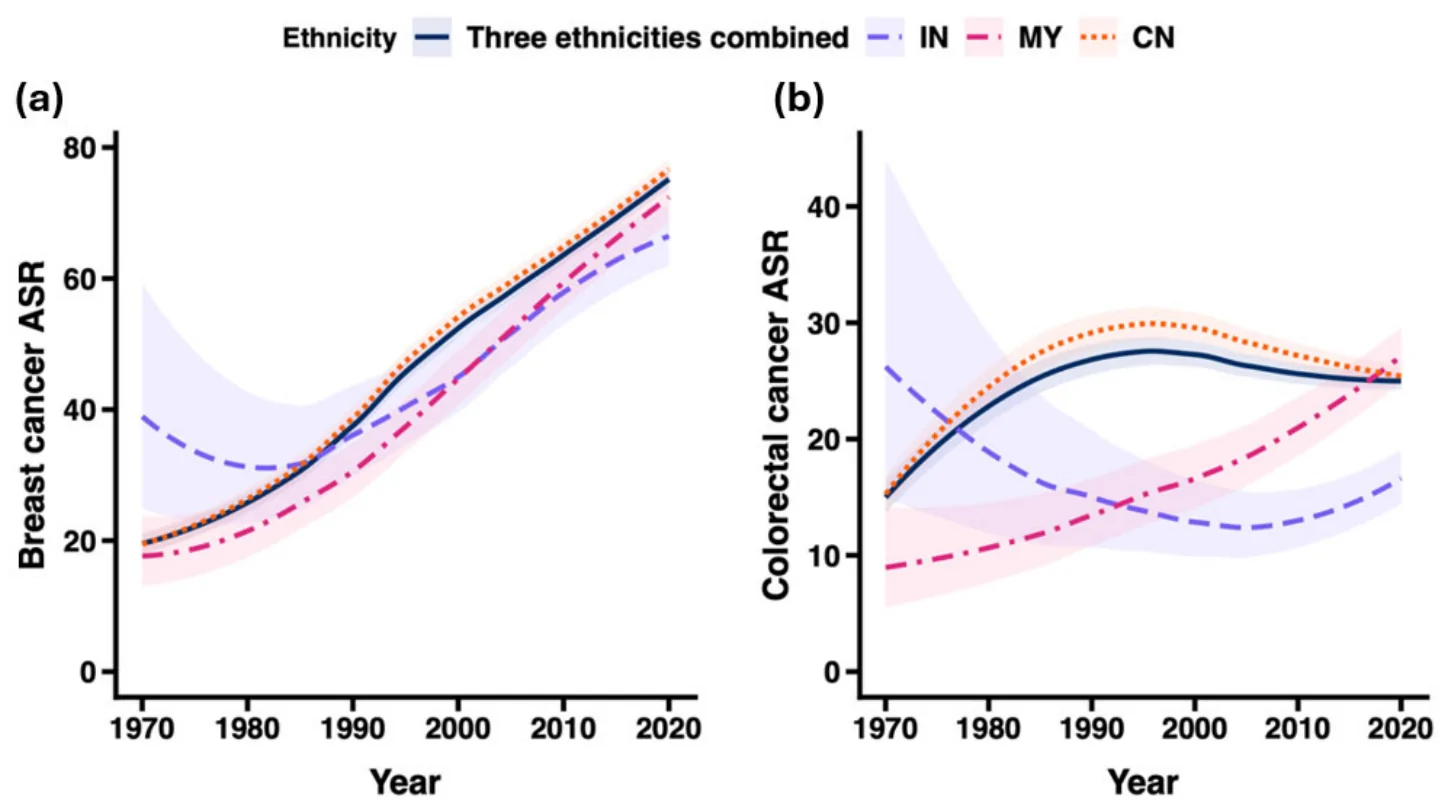
Age-standardised incidence rates (ASR) of breast (**a**) and colorectal (**b**) cancer among females aged 25 to 84 years in Singapore, by ethnicity, 1968 to 2022. ASR are per 100,000 person-years, directly standardised to the Segi (1960) world standard population. The weight for each of the twelve 5-year age groups from 25 to 29 through 80 to 84 was its standard population divided by the full Segi total of 100,000 (weights not rescaled to the restricted age range). Rates were calculated on the complete age–period grid, with person-years for every cell and age–period cells absent from the case extract set to zero cases. Counts suppressed as “<5” were set to 3. Year refers to the midyears of 5-year periods. CN = Chinese, IN = Indian, MY = Malay, Three ethnicities combined = CN + IN + MY. Curves were obtained by Loess smoothing (span = 0.75, weighted by case counts) of the period-specific ASR values. Shading shows Loess-smoothed 95% gamma confidence limits (Fay and Feuer method) of the period-specific ASR values.

As shown in the smoothed trends, BC ASR increased overall across all three ethnic groups. Among Chinese women, BC ASR rose from 1970 onwards, and among Malay women from 1975 onwards (Figure 1, Table S1). Among Indian women, BC ASR declined from 1970 to about 1980–1985, with wide CIs. Sensitivity analysis indicated that early ASR trends for both BC and CRC among Indian women were affected by the imputation value used in cells with <5 counts, and the decline in BC ASR among Indian women did not emerge when suppression values were imputed to a count of 1 instead of 3 (Figure S1).

CRC ASR increased among Chinese women from the 1970s to the 1990s before declining (Figure 1, Table S1). In Malay women, CRC ASR increased from 8.4 per 100,000 in 1970 to 26.9 in 2020 (Table S1), apart from a dip in 1985. Among Indian women, CRC ASR increased from the mid-2000s onwards, but the decline from 1970 to the mid-2000s was similarly sensitive to suppression-value imputation.

### 3.2. Age-Specific Rates of Breast and Colorectal Cancer

**Figure 2:**
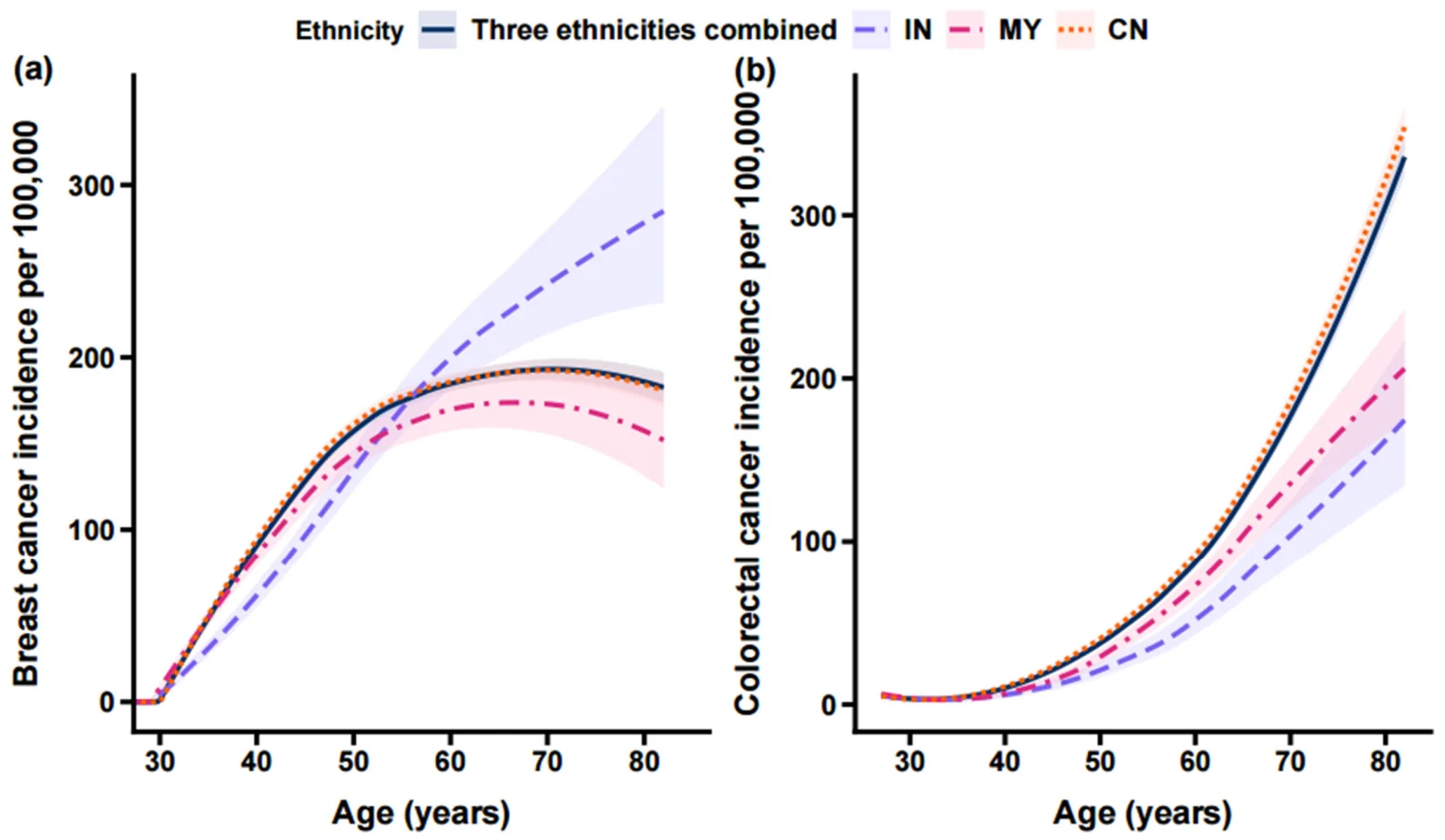
Age-specific incidence rates of breast (**a**) and colorectal (**b**) cancer among females aged 25 to 84 years in Singapore, by ethnicity, pooled over 1968 to 2022. Rates are per 100,000 person-years, calculated from cases and person-years summed across all 5-year periods within each 5-year age group. Person-years were taken from the complete age–period grid, and age–period cells absent from the case extract were set to zero cases. Counts suppressed as “<5” were set to 3. Age refers to the midyears of 5-year age groups. CN = Chinese, IN = Indian, MY = Malay, Three ethnicities combined = CN + IN + MY. Curves were obtained by Loess smoothing (span = 0.75, weighted by case counts) of the age-specific rates. Shading shows Loess-smoothed 95% exact Poisson confidence limits of the age-specific rates. The breast cancer panel is displayed from age 30.

Among women younger than 49 years, smoothed trends show BC incidence generally increased with age in all three ethnic groups and was highest among Chinese women, followed by Malay and Indian women (Figure 2). In the age-specific descriptive rates, BC incidence began to flatten from around age 50 and plateaued by about age 60 among Chinese and Malay women (declining slightly after about age 70 in Malay women) but continued to rise among Indian women (Figure 2). CRC incidence rates were generally highest among Chinese women, followed by Malay and Indian women (Figure 2).

**Figure 3:**
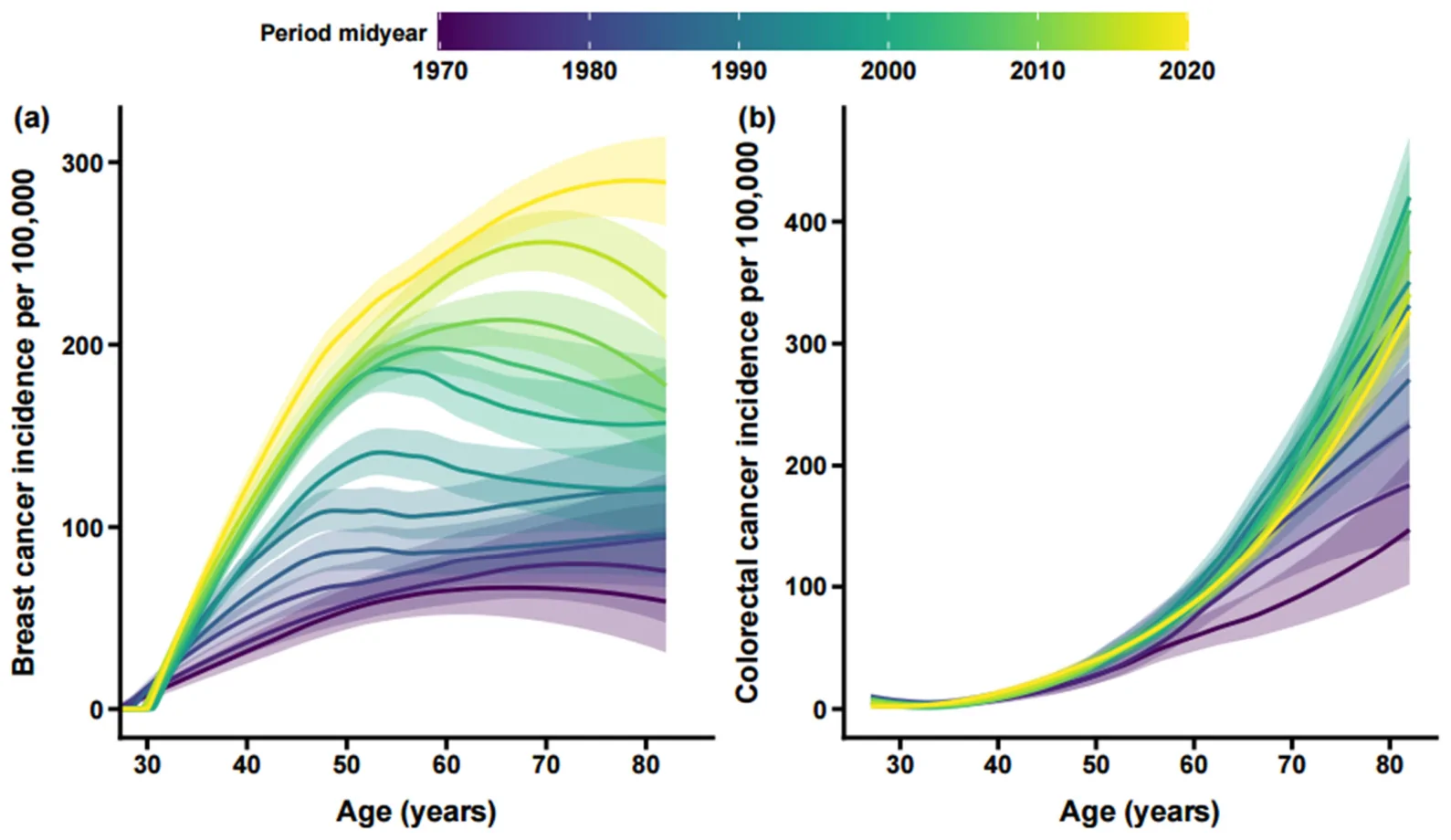
Age–incidence relationship for breast (**a**) and colorectal (**b**) cancer in females aged 25 to 84 years in Singapore, for the three ethnic groups combined (Chinese + Indian + Malay), by 5-year diagnosis period from 1968 to 2022. Rates are per 100,000 person-years, calculated for each period from cases and person-years in the complete age–period grid. Age–period cells absent from the case extract were set to zero cases, and counts suppressed as “<5” were set to 3. Each curve represents one period, coloured by period midyear. Age refers to the midyears of 5-year age groups. Curves were obtained by Loess smoothing (span = 0.75, weighted by case counts) of the age-specific rates within each period. Shading shows Loess-smoothed 95% exact Poisson confidence limits of the age-specific rates. The breast cancer panel is displayed from age 30 years.

Period-specific trends for all women revealed that BC incidence curves increased and steepened in recent periods for both women aged <49 years and those aged 49 years and older, while pre-2000 increases in CRC incidence were most prominent among older women (Figure 3).

### 3.3. Component Effects Within Different Ethnicities

The full APC models had the lowest AIC among the models compared for both BC (1442.7) and CRC (1073.2) in the combined population, as well as for Chinese women (Table 1). Among Indian women, adding period effects to the age–cohort model did not significantly improve BC model fit (*p* = 0.404). The age–cohort model was therefore adequate for BC among Indian women. However, the sensitivity analysis revealed that nested model comparisons were sensitive to imputation values for Indian CRC (Table S2), so conclusions regarding the statistical significance of period and cohort effects cannot be drawn reliably for this subgroup. For Indian BC, these comparisons were robust to the imputation value (Table S2).

**Figure 4:**
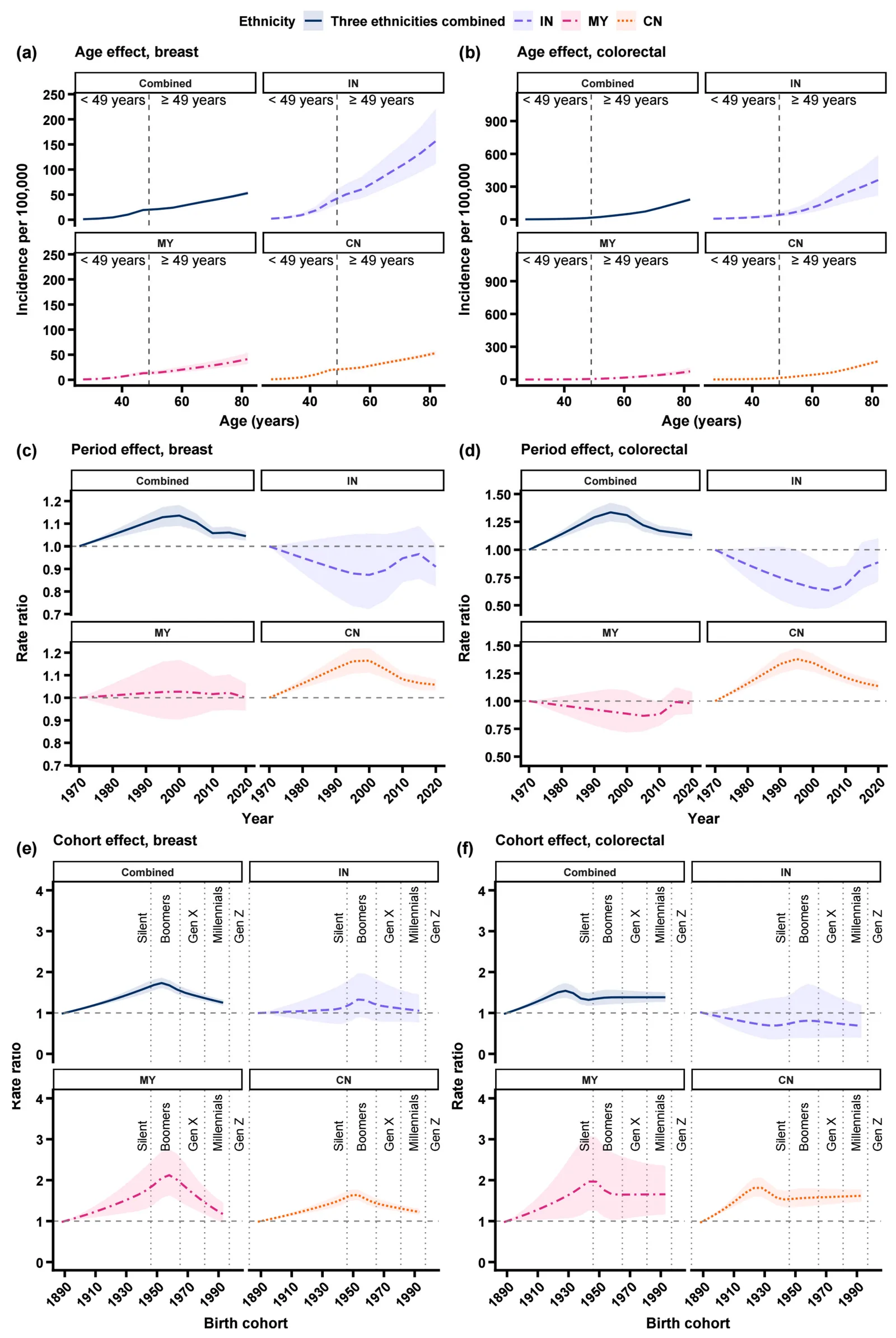
Age, period and cohort effects on breast and colorectal cancer incidence among females aged 25 to 84 years in Singapore from 1968 to 2022, for the three ethnic groups combined and by ethnicity. Panels (**a**,**c**,**e**) show breast cancer; panels b, d and f show colorectal cancer. Effects were estimated from Poisson age–period–cohort models with natural-spline terms (Epi::apc.fit), fitted to the complete 12 × 11 age–period grid for each cancer and ethnic group, with person-years for every cell, age–period cells absent from the case extract set to zero cases, and counts suppressed as “<5” set to 3. Panels (**a**,**b**) show age effects (incidence per 100,000 person-years); panels (**c**,**d**) show period rate ratios and panels (**e**,**f**) cohort rate ratios (non-linear components, with net drift removed). Dashed vertical lines in a-b mark the boundary between the <49- and ≥49-year age groups; dashed horizontal lines in c-f mark a rate ratio of 1; dotted vertical lines in e-f mark generation boundaries. The reference period was 1970 (the first period midyear) and the reference cohort was 1890 (approximately the earliest cohort; earliest cohort midyear 1888). Age, year and birth cohort refer to 5-year midyears. Shading indicates 95% confidence intervals. CN = Chinese, IN = Indian, MY = Malay, Combined = CN + IN + MY. Cohort rate ratios are displayed on a truncated scale (0–4), and the cohort axis is shown from 1888 to 2000.

Among Malay women, adding period effects did not significantly improve model fit for BC incidence (*p* = 0.959) or CRC (*p* = 0.296). The deviance reduction for Malay CRC per degree of freedom was smaller for period effects than for cohort effects (1.57 versus 5.10), and the period rate-ratio confidence intervals for Malay CRC largely included 1.0 (Figure 4d). For both cancers in Malay women, the *p*-values and AIC supported the age–cohort model over the full APC model (BC: 801.6 versus 807.2; CRC: 672.3 versus 673.6). Dispersion ratios indicated mild overdispersion in the Indian and Malay strata (χ^2^/df 1.05–1.26) and more substantial overdispersion in the Chinese and combined groups (χ^2^/df 1.53–3.82; Table 1). For Indian CRC, in which 53 (40.2%) of the 132 age–period cells were suppressed, χ^2^/df ranged from 1.05 to 1.43 across imputation values of 1 to 4 (Table S2).

According to the tests of period effects conditional on cohort effects (Table 1), period effects on BC were significant only among Chinese women (*p* < 0.05) among the three ethnic groups. Model-estimated age-specific incidence of both BC and CRC increased with biological age in the reference cohort across all ethnicities once period effects have been accounted for (Figure 4a,b). For CRC, period effects were significant among Chinese women (*p* < 0.001). Among Indian women, the period effect given cohort was significant with imputation values of 3 (*p* < 0.05) and 4 (*p* < 0.01) but not 1 (*p* = 0.684) or 2 (*p* = 0.096), and the cohort effect given period was not significant at any imputation value (*p* = 0.257 to 0.915; Table S2). The data therefore provide no consistent evidence of a cohort effect for Indian CRC, and evidence for a period effect depends on the imputation assumption. As shown in Figure 4c,d, the period rate ratio (P-RR) for both BC and CRC peaked in the mid-to-late 1990s among Chinese women before declining. Among Indian women, P-RR for CRC fell from 1970 to the mid-2000s before rising again.

According to the tests of cohort effects conditional on period effects, cohort effects on BC were significant across all three ethnic groups, whereas cohort effects on CRC were significant among Chinese and Malay women (Table 1). As shown in Figure 4e, the C-RR for BC peaked among the Baby Boomer generation across all ethnic groups. Among Chinese women, the C-RR for CRC (Figure 4f) increased with later cohorts until those born in the 1920s, after which it declined and then plateaued. Among Malay women, CRC C-RRs rose across successive cohorts up to the Baby Boomer generation, after which they declined and stabilised (Figure 4f).

**Figure 5:**
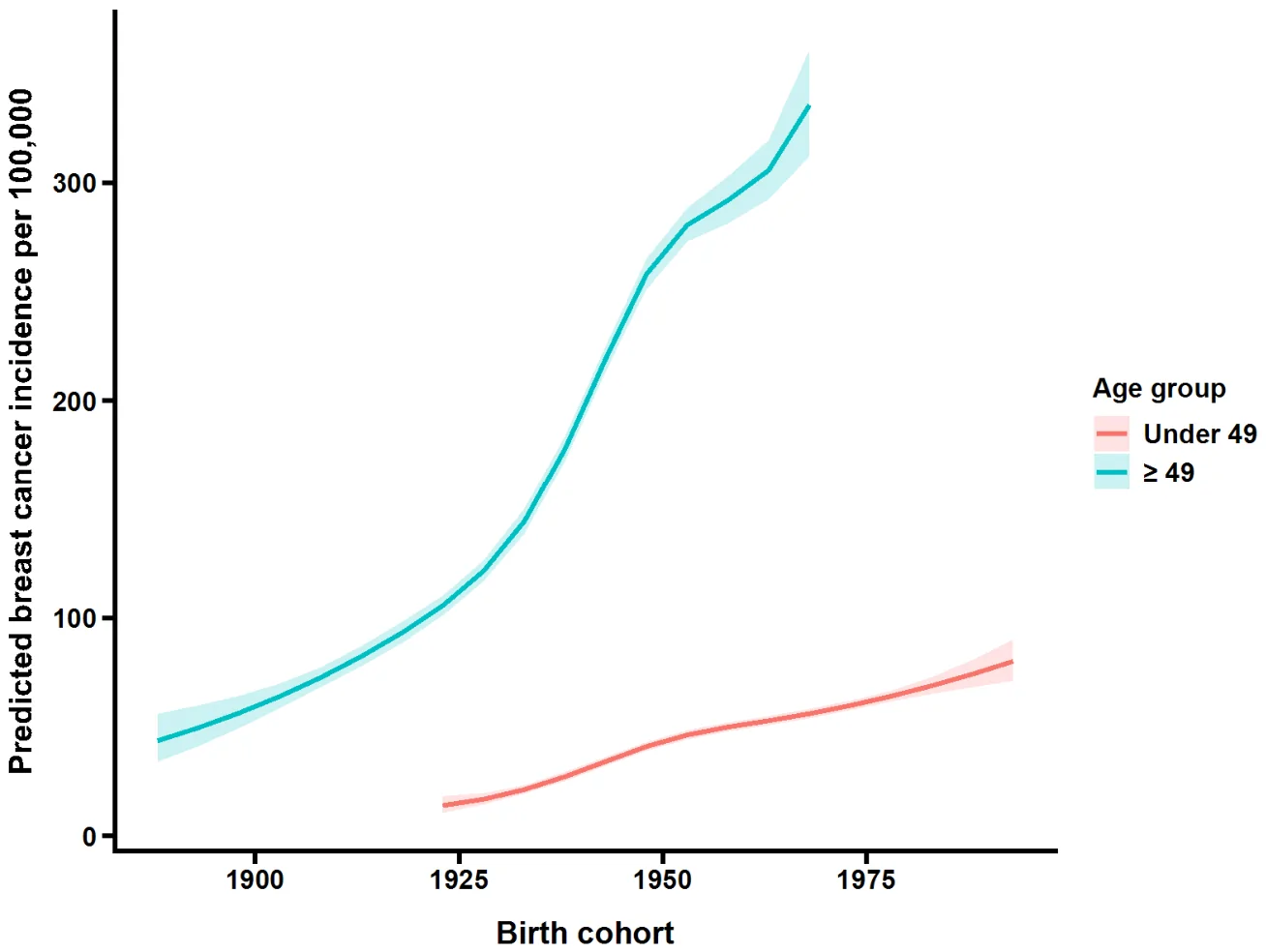
Overall predicted breast cancer incidence by birth cohort among women in age groups below 49 years and 49 years and above in Singapore, for the three ethnic groups combined (Chinese, Indian and Malay). Predicted incidence per 100,000 person-years was derived from a quasi-Poisson generalised linear model (log link, with log person-years as offset) fitted to the complete age–period grid, with age–period cells absent from the case extract set to zero cases and counts suppressed as “<5” set to 3. The model included a natural cubic spline of birth cohort (5 degrees of freedom), a two-level age-group term (age-band midyear below 49 vs. 49 and above), the interaction between the cohort spline and the age-group term, and a natural cubic spline of age (5 degrees of freedom). Predictions were made at the median age-band midyear of each age group (37 and 67 years) and only for birth cohorts represented in that group’s age–period cells. Birth cohort refers to 5-year midyears (period midyear minus age midyear). Shading indicates 95% confidence intervals, calculated on the log scale from standard errors that incorporate the estimated dispersion, using the t-distribution with the model’s residual degrees of freedom, and back-transformed.

The age-group (<49 versus ≥49 years) by birth-cohort interaction significantly improved model fit overall (F-statistic = 3.7718, *p* = 0.003365). The interaction also improved fit among Chinese women (F-statistic = 3.7686, *p* = 0.003385), but not among Indian (F-statistic = 1.9768, *p* = 0.08726) or Malay women (F-statistic = 0.6365, *p* = 0.6723). Predicted BC incidence was generally higher among women aged ≥49 years than among those aged <49 years and increased more steeply from cohorts born in the 1930s onwards (Figure 5).

## 4. Discussion

Using 55 years of national registry data, we found contrasting temporal patterns for BC and CRC among Singaporean women. BC incidence increased mainly along a linear drift, beyond which non-linear cohort effects were more strongly supported than non-linear period effects, whereas the relative contributions of period and cohort effects to CRC differed by ethnicity. The side-by-side analysis of both cancers highlights ethnic heterogeneity that is obscured by national aggregate trends.

### 4.1. Breast Cancer Incidence: Stronger Support for Cohort than Period Curvature

Our analyses show that cohort-associated trends have persisted since the study by Sim et al. [22]. The rise in BC incidence with age among women younger than 49 years, with incidence flattening from around age 50 among Chinese and Malay women, aligns with long-established observations explained by the cessation of ovarian hormone production [41]. The cohort-related rise in BC incidence coincides with historical declines in parity and increases in adiposity among Baby Boomer women [42,43,44,45,46], whose entry into adulthood coincided with socioeconomic changes and policies such as “Stop at Two” (1972) which were associated with later marriage and delayed childbearing in younger cohorts [47,48,49]. However, the contributions of these factors to the observed incidence trends cannot be determined from this analysis. In addition, because the age-specific rates in Figure 2 pool all periods and cohorts, and older ages are observed mainly in earlier birth cohorts, the age–incidence shapes may not reflect age effects alone.

Rising age at first birth and declining age at menarche may steepen the age-incidence curve for women younger than 49 years in Singapore [50,51,52]. Predicted BC incidence showed different trajectories across age groups (<49 and ≥49 years), with a more pronounced increase among older women in the observable birth cohorts. However, because the two age groups are observed over different, only partly overlapping ranges of birth cohorts (50–84 years: cohorts born c.1888–1968; 25–49 years: c.1923–1993) and absolute incidence differs between them, the relative strength of cohort effects cannot be compared reliably.

Like Singapore, Sweden and Shanghai have experienced declines in total fertility rate (TFR), defined as the number of births per woman; differences in the magnitude of these declines are reflected in their differing BC temporal trends. Sweden and Singapore are advanced economies [53] with relatively small populations (10.6 million and 6.1 million, respectively), whereas Shanghai and Singapore are both urban, population-dense cities with Chinese ethnic majorities. Singapore appears to have experienced the largest rate of decline in TFR among the three settings since 1960 [54]. Singapore’s initially higher TFR in the 1960s compared with Sweden may have contributed to its lower BC ASR (ASR values for Shanghai and Sweden were obtained from the CI5 database, where incidence rates were standardised with the Segi, 1960, world standard population [31,55]), and its subsequent decline in TFR may have contributed to rising BC incidence, although fertility trends alone cannot explain the observed patterns [55]. Notably, Shanghai’s BC ASR remains lower than Singapore’s despite having the lowest TFR. This could be due to potentially differing degrees of westernisation, diet and lifestyle in the two cities, despite ethnic similarities [56]. If pro-natalist policies continue to have limited effect on TFR, we speculate that Singapore’s BC ASR may continue to rise [47].

In Chinese women, rising BC incidence could be influenced in part by period-related factors such as increasing uptake of BC screening [57,58]. Although most of the temporal increase in BC incidence was captured by the non-identifiable linear drift, the identifiable nonlinear cohort component contributed more to model improvement than the corresponding nonlinear period component. This supports a stronger association with cohort curvature, without attributing the overall secular increase specifically to cohort-related factors. Net linear drift explained the most deviance per additional parameter, followed by cohort effects. Non-linear cohort effect contributions rose until Baby Boomer women born in the 1950s. This timing coincides with Singapore’s steepest decline in total fertility rate between 1960 and 1978, which affected women in their peak childbearing years during that period (approximately those born between the mid-1920s and 1950s). However, we cannot establish that reduced parity accounts for the cohort pattern, because parity and other reproductive exposures were not measured in this study. Declining detrended C-RRs in the more recent cohorts indicate a slower increase than the overall linear trend rather than lower absolute risk and partly reflect the limited age ranges over which these cohorts have been observed.

The age-group by birth-cohort interaction did not significantly improve model fit among Indian or Malay women. For Malay women, this may reflect differences in reproductive or other risk-factor profiles, whereas for Indian women the smaller number of cases limits statistical power. Neither explanation could be evaluated directly in the present analysis [45,51,59].

The available adiposity data do not clearly explain the divergent age–incidence curve among Indian women aged 49 years and older. Malay and Indian women have been reported to share a similar predicted BMI trajectory [44]. These ethnic differences in age–incidence patterns may reflect an intermingling of age and cohort risk factors, and possibly differences in dominant tumour subtypes according to hormone-receptor status (ER/PR) [60].

While cohort-related factors have been more strongly associated with temporal BC incidence trends thus far, period-related factors may become more influential in the future. For example, changes in hormone replacement therapy prescribing could alter population-level exposure patterns. The hormone replacement therapy–BC risk association is formulation-dependent, suggesting that the extent of future period effects will depend on how commonly each formulation is prescribed, with oestrogen-only treatments carrying lower risk than oestrogen–progestogen therapy (EPT) [61].

### 4.2. Differences in Colorectal Cancer Temporal Patterns

Unlike BC, CRC incidence continued to increase with age, consistent with the cumulative effects of biological ageing and long-term exposure to colorectal carcinogenic factors [62]. In crude age-specific rates, the smallest increase in CRC rates with age was observed among Indian women. This pattern may be partly related to dietary differences, although the ecological design does not permit attribution to specific dietary behaviours [63,64].

While Burkill et al. [23] compared cohort and period effects on CRC between different ethnicities, they did not conduct ethnically stratified APC analyses within the female population specifically. APC analyses from Chen et al. [24] up to the year 2019 were concentrated on cohort effects in the overall Singapore population, with an emphasis on comparing colon and rectal cancer and on early-onset CRC. Our analyses suggest that cohort effects were more strongly supported among Malay women, whereas both period and cohort effects contributed to model fit among Chinese women. CRC ASR trends and cohort effects identified in the present study were largely consistent with previous findings, apart from some variation among Indian women. Burkill et al. [23] found that period effects were not statistically significant in the overall Indian population, whereas our corresponding finding for Indian women depended on the imputation value. Differences in study populations and methods, and the sensitivity of early trends and CRC nested-model comparisons among Indian women to suppressed-count imputation, call for careful interpretation.

The increasing CRC cohort rate ratios across cohorts born before the mid-20th century coincide with broader dietary and lifestyle transitions, although the contributions of these exposures cannot be established from our analysis [65,66]. CRC ASR rose overall among Malay women, whereas it rose until the 1990s and then declined among Chinese women. These differing trends may reflect ethnic variation in risk-factor profiles, healthcare utilisation, or other influences that cannot be clarified using aggregate incidence data [44,67,68].

Among Chinese women, CRC cohort rate ratios increased until cohorts born in the 1920s and then declined and plateaued. Both BC and CRC showed P-RR peaks in the mid-to-late 1990s, followed by declines. These patterns may partly reflect changes in screening uptake, diagnostic practices, and healthcare utilisation [57]. However, period effects can capture multiple concurrent influences. Our analysis cannot isolate the contribution of screening. Similarly, the absence of a distinct period effect following the introduction of the national CRC screening programme in 2011 does not necessarily mean the programme has limited impact. Only the last one or two five-year periods follow the 2011 launch, which limits the ability of the spline to resolve any such effect. Screening-related changes may emerge gradually and depend on population coverage, participation, and other concurrent temporal influences. Individual-level screening histories and programme participation data would be needed to assess these explanations directly.

### 4.3. Strengths, Limitations and Implications

To aid interpretation, we used widely recognised generational labels (Silent Generation, Baby Boomers, Gen X, Millennials) purely as descriptive shorthand for adjacent birth cohorts, to make cohort patterns easier for non-specialist readers to relate to historical context such as the “Stop at Two” policy. These boundaries were defined in Western settings and may not match Singapore’s demographic history, and all inferences rest on the birth-cohort analysis, not on the generational categories. The labels therefore add no analytical information beyond the birth-cohort estimates themselves.

A limitation of our study is the ecological design, which prevents linking individual-level exposures to individual cancer outcomes and limits causal interpretation. Furthermore, period and cohort effects represent aggregate temporal associations rather than specific causal mechanisms. Without individual-level information on screening participation, reproductive history, adiposity, and other exposures, the contributions of these factors to observed incidence trends cannot be established. Another limitation is the relatively small number of cases among Indian women, which reduced the precision of estimates for this group. As such, trends observed in this group should be interpreted carefully. Sensitivity analyses showed that assumptions regarding the imputation of suppressed counts affected the earlier temporal trends among Indian women and the nested-model comparisons for Indian CRC. Other limitations are the restriction to ages 25 to 84, the possible changes in ethnic classification over time, the identifiability constraint inherent to APC models, and the use of quasi-Poisson models without a period term for the age-group analysis. The age at menopause varies between individuals and may differ across ethnic groups. These age-based categories may thus not accurately reflect menopausal status, and observed differences in incidence trends between age groups cannot be directly attributed to menopause.

Although this study is specific to Singapore, the findings may be relevant to other rapidly transitioning, multi-ethnic populations. The contrasting temporal patterns of BC and CRC demonstrate how national trends can obscure subgroup differences and illustrate the value of applying ethnicity-stratified APC analyses to population-based registry data. By applying similar APC frameworks to their own registry data, other countries could generate comparable evidence on whether societal transitions are associated differentially with cancer types and population subgroups.

## 5. Conclusions

Our findings show that, beyond linear drift, nonlinear cohort effects better explain departures of BC incidence from its linear trend in Singaporean women than period curvature. Birth-cohort associations with BC incidence also varied between younger and older age groups, although differences in observable cohort ranges limit direct comparisons of their relative magnitude. Temporal patterns of CRC incidence, meanwhile, diverged by ethnicity. Generational risk exposures should therefore be examined directly and be considered when interpreting current policy needs. Ethnic differences in the temporal correlates of CRC incidence should also be considered, while recognising that these differences may reflect both risk-factor distributions and unequal screening, healthcare utilisation and diagnostic ascertainment. For Indian women, however, the relative contributions of period and cohort effects to CRC incidence remained uncertain because model comparisons were sensitive to suppressed-count imputation. As Singapore’s population continues to age, ethnically stratified population studies such as this can help anticipate which groups will bear a disproportionate cancer burden and inform responsive health policy.

## Figures and Tables

**Table 1:** Goodness-of-fit measures and nested-model comparisons for age–period–cohort (APC) analyses of breast and colorectal cancer incidence among females aged 25 to 84 years in Singapore from 1968 to 2022, for the three ethnic groups combined and by ethnicity. Models were fitted to the complete 12 × 11 age–period grid for each cancer and ethnic group, with person-years for every cell. Age–period cells absent from the case extract were set to zero cases, and counts suppressed as “<5” were set to 3. The reference period was 1970 and the reference cohort was 1890 ^1^.

			Breast					Colorectal				
Ethnicity	Model	Effect Tested	*AIC*	*df*	*p*	*Dev*	*χ^2^/df*	*AIC*	*df*	*p*	*Dev*	*χ^2^/df*
**Combined**	Age	-	7565.6	127	-	6607.1	3.82	1409.7	127	-	539.2	1.57
	Age + Drift	Drift	1894.7	126	<0.001	934.2		1361.8	126	<0.001	489.2	
	Age + Cohort	Cohort given drift	1474.3	123	<0.001	507.8		1153.8	123	<0.001	275.3	
	Age + Period + Cohort	Period given cohort	1442.7	120	<0.05	470.2		1073.2	120	<0.001	188.7	
	Age + Period	Cohort given period	1721.7	123	<0.001	755.2		1153.5	123	<0.001	275	
**Chinese**	Age	-	6577.8	127	-	5642.7	3.51	1479.5	127	-	628.9	1.53
	Age + Drift	Drift	1772.1	126	<0.001	835		1452.1	126	<0.001	599.6	
	Age + Cohort	Cohort given drift	1422.4	123	<0.001	479.3		1142.4	123	<0.001	283.8	
	Age + Period + Cohort	Period given cohort	1385.1	120	<0.05	436		1049.3	120	<0.001	184.7	
	Age + Period	Cohort given period	1604.7	123	<0.001	661.6		1170.4	123	<0.001	311.8	
**Indian**	Age	-	883.1	127	-	317.9	1.22	581.5	127	-	174.9	1.05
	Age + Drift	Drift	721.5	126	<0.001	154.3		582.5	126	0.381	173.9	
	Age + Cohort	Cohort given drift	714.8	123	<0.05	141.6		579.7	123	0.051	165	
	Age + Period + Cohort	Period given cohort	716.9	120	0.404	137.7		573	120	<0.05	152.3	
	Age + Period	Cohort given period	723.7	123	<0.05	150.5		570	123	0.452	155.3	
**Malay**	Age	-	1649.7	127	-	1003.8	1.26	844.6	127	-	327.6	1.12
	Age + Drift	Drift	873.3	126	<0.001	225.3		679.1	126	<0.001	160.1	
	Age + Cohort	Cohort given drift	801.6	123	<0.001	147.6		672.3	123	<0.05	147.3	
	Age + Period + Cohort	Period given cohort	807.2	120	0.959	147.2		673.6	120	0.296	142.6	
	Age + Period	Cohort given period	866.7	123	<0.001	212.7		682.9	123	<0.01	157.9	

^1^ AIC is the Akaike information criterion; df is the residual degrees of freedom and Dev the residual deviance of each model; the full age–period–cohort model has 120 residual degrees of freedom in every group because each group has 132 cells. The *p* column gives Benjamini–Hochberg-adjusted *p*-values from quasi-likelihood F-tests of nested models. The deviance difference per degree of freedom was divided by a dispersion factor, defined as the Pearson chi-squared divided by the residual degrees of freedom of the full age–period–cohort model (χ^2^/df, shown once for each cancer–ethnicity block) and floored at 1. *p*-values were obtained from the F distribution, using the test degrees of freedom and the residual degrees of freedom of the full model. They were adjusted for multiple testing across all model comparisons for both cancers and all ethnic groups, including the test of period given drift, which is not displayed. Age + Drift is compared with Age, Age + Cohort with Age + Drift, and Age + Period + Cohort with Age + Cohort, so a *p*-value below 0.05 for Age + Period + Cohort indicates that adding period to the Age + Cohort model significantly improved fit. Age + Period is compared with the full age–period–cohort model, which tests the cohort effect given period. These *p*-values differ from the unadjusted Poisson likelihood-ratio *p*-values (as reported in Sim et al. [22]). “-” = not applicable.

## Data Availability

The data that support the findings of this study are available from the National Registry of Diseases Office (NRDO), Singapore, but restrictions apply to the availability of these data, which were used with approval for the current study, and so are not publicly available. Data are however available from NRDO upon reasonable request (https://www.nrdo.gov.sg/data-request/data-request-process/, accessed on 29 October 2026).

## Supplementary Materials

The following supporting information can be downloaded at: https://www.mdpi.com/article/10.3390/cancers18213538/s1, Figure S1: Sensitivity of age-standardised incidence rates (ASR) to the value used to replace suppressed “<5” counts, in females aged 25 to 84 in Singapore, 1968 to 2022; Table S1: Number of cases, population person-years, and age-standardised incidence rates (ASRs) of breast and colorectal cancer among women aged 25-84 years in Singapore, by ethnicity and five-year period, 1968–2022; Table S2: Sensitivity analysis of nested APC model comparisons and dispersion ratios among Indian women using different imputed values (1, 2, 3 and 4) for suppressed counts.
