# Supplementary material for: Comparative Age-Period-Cohort Analysis Reveals Divergent Temporal Trajectories of Breast and Colorectal Cancer Among Women in Singapore
Source: Cancers (Basel). 2026 Nov 3;18(21):3538. doi: 10.3390/cancers18213538 (PMC13761586; doi:10.3390/cancers18213538)
Supplement: Supplementary file 1 [file cancers-18-03538-s001.zip › cancers-4619281-supplementary.pdf]

Figure S1. Sensitivity of age-standardised incidence rates (ASR) to the value used to replace suppressed "<5" counts, in females aged 25 to 84 in Singapore, 1968 to 2022. Suppressed "<5" counts were set to 1, 2, 3 or 4. Panels a, c, e and g show breast cancer and panels b, d, f and h show colorectal cancer, for imputed values of 1, 2, 3 and 4 respectively. Age-period cells absent from the case extract were set to zero cases in all scenarios. ASRs per 100,000 person-years were directly standardised to the Segi world standard population (weights = standard population / 100,000), with gamma-based 95% confidence intervals. Year refers to the midyears of 5-year periods. CN=Chinese, IN=Indian, MY=Malay, three ethnicities combined=CN+IN+MY. Curves and confidence limits were smoothed separately with Loess (span = 0.75), weighted by the number of cases in each period for each cancer and ethnic group. Shading indicates the smoothed 95% confidence intervals. Imputed value 3 was used in the main analysis **(Figure 1)**.

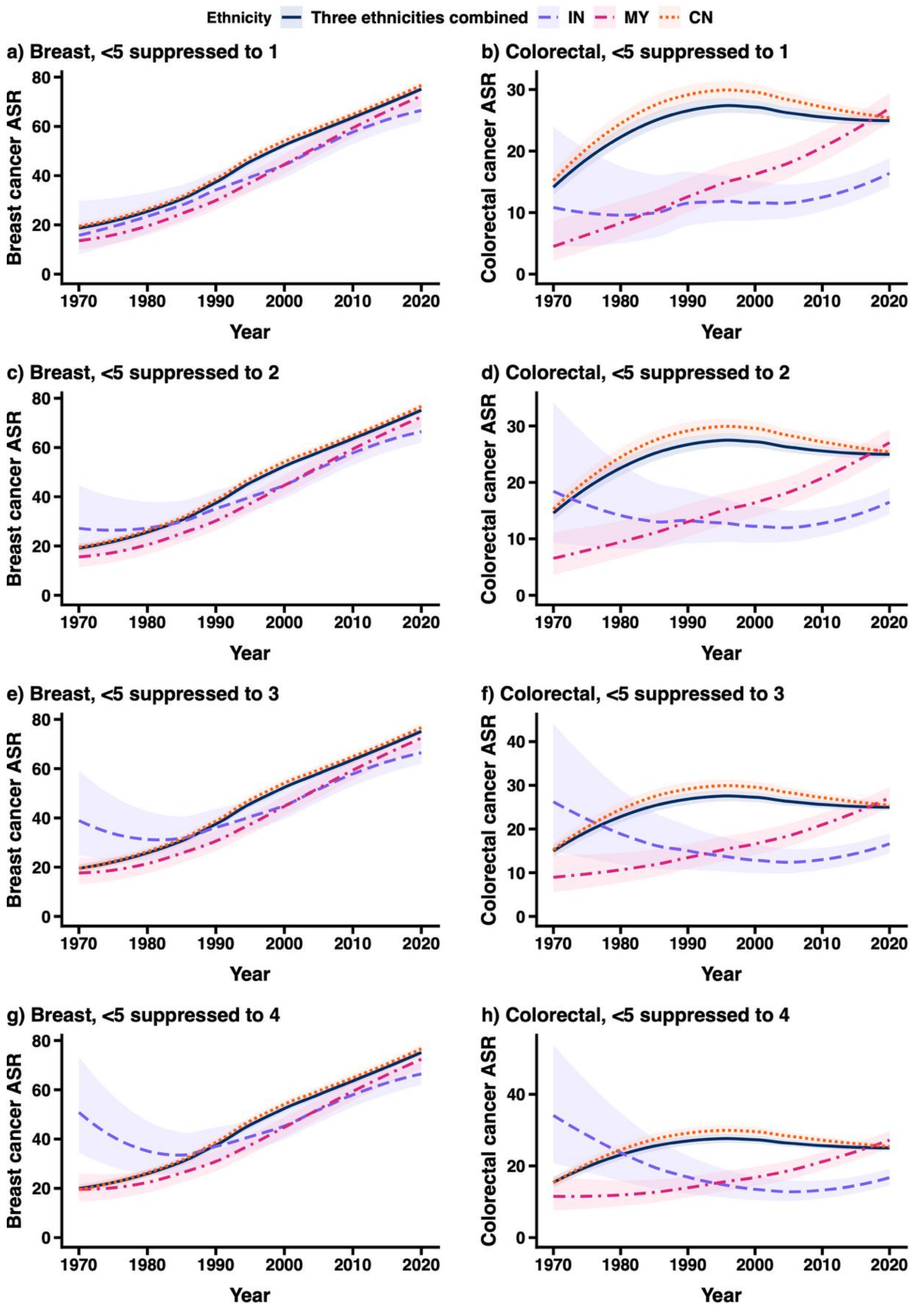

Table S1. Number of cases, population person-years, and age-standardised incidence rates (ASRs) of breast and colorectal cancer among women aged 25-84 years in Singapore, by ethnicity and five-year period, 1968-2022. CN = Chinese; IN = Indian; MY = Malay; Combined = CN + IN + MY. Period midyear is the midpoint of each five-year period. Population is expressed as person-years. Age-standardised rates (ASR) are per 100,000 person-years, calculated by direct standardisation of pooled age-specific rates to the Segi world standard population (each age group's standard population divided by the Segi total of 100,000). Only ages 25-84 were analysed, so ASRs assume no cases outside this range and may be marginally lower than all-age ASRs. The 95% confidence intervals were calculated using the gamma method. Counts suppressed as "<5" were imputed as 3.

Ethnicity	Period	Person-years	Cases	Crude rate per 100,000	ASR per 100,000	ASR 95% CI	Cases	Crude rate per 100,000	ASR per 100,000	ASR 95% CI
Chinese	1968-1972	1582501	552	34.9	19.3	17.7-21.0	436	27.6	15.5	14.0-17.0
Chinese	1973-1977	1887820	734	38.9	22.2	20.6-23.9	651	34.5	19.8	18.3-21.4
Chinese	1978-1982	2275684	1044	45.9	27	25.3-28.7	982	43.2	25.1	23.5-26.8
Chinese	1983-1987	2769000	1451	52.4	31.3	29.7-33.0	1288	46.5	27.2	25.7-28.8
Chinese	1988-1992	3266822	2181	66.8	38.9	37.3-40.7	1633	50	29.1	27.6-30.6
Chinese	1993-1997	3760800	2954	78.5	44.1	42.4-45.7	1938	51.5	30.1	28.8-31.5
Chinese	1998-2002	4261200	4599	107.9	57.1	55.4-58.8	2301	54	29.5	28.2-30.7
Chinese	2003-2007	4617100	5521	119.6	59.4	57.9-61.1	2712	58.7	28.9	27.8-30.1
Chinese	2008-2012	5053162	6756	133.7	63.4	61.8-64.9	3068	60.7	26.5	25.5-27.5
Chinese	2013-2017	5437851	8488	156.1	70.5	68.9-72.0	3589	66	26	25.1-26.8
Chinese	2018-2022	5782747	10236	177	76.9	75.4-78.5	4152	71.8	25.6	24.8-26.4
Indian	1968-1972	89862	36	40.1	39.8	25.4-60.5	20	22.3	26.2	14.7-44.2
Indian	1973-1977	111369	45	40.4	34.2	23.4-49.3	24	21.5	20.6	12.1-33.7
Indian	1978-1982	142354	54	37.9	27.4	20.0-37.8	34	23.9	22	14.6-32.7
Indian	1983-1987	194300	90	46.3	33.1	26.1-41.9	31	16	15.3	10.0-22.7
Indian	1988-1992	254962	120	47.1	34.7	28.4-42.3	38	14.9	12.1	8.2-17.2
Indian	1993-1997	315400	191	60.6	40.2	34.4-46.8	64	20.3	15.9	12.1-20.7
Indian	1998-2002	382400	291	76.1	47.1	41.7-53.2	73	19.1	13.1	10.2-16.7
Indian	2003-2007	439800	370	84.1	48.3	43.4-53.8	76	17.3	11	8.6-13.9
Indian	2008-2012	529612	543	102.5	58.3	53.3-63.6	114	21.5	13.1	10.7-15.9
Indian	2013-2017	571836	711	124.3	65	60.3-70.1	166	29	15.1	12.8-17.7
Indian	2018-2022	600755	834	138.8	65.5	61.1-70.2	214	35.6	16.3	14.2-18.8
Malay	1968-1972	238264	68	28.5	19.1	14.3-25.5	24	10.1	8.4	5.0-13.5
Malay	1973-1977	280934	75	26.7	16.7	12.9-21.5	35	12.5	9.5	6.4-13.8
Malay	1978-1982	338036	113	33.4	21.9	17.8-26.8	54	16	12.6	9.3-16.9
Malay	1983-1987	415100	155	37.3	23.2	19.5-27.4	58	14	10	7.5-13.2
Malay	1988-1992	502251	260	51.8	32.9	28.8-37.4	94	18.7	13.3	10.7-16.4
Malay	1993-1997	580700	350	60.3	36.3	32.4-40.5	129	22.2	15.7	13.1-18.8
Malay	1998-2002	640700	514	80.2	43.4	39.5-47.5	166	25.9	16.9	14.3-19.7
Malay	2003-2007	688500	723	105	54	49.9-58.3	207	30.1	18.2	15.7-21.0
Malay	2008-2012	758182	910	120	58	54.2-62.1	297	39.2	20.5	18.1-23.0
Malay	2013-2017	832886	1163	139.6	66.8	63.0-70.9	436	52.3	24.6	22.2-27.1
Malay	2018-2022	907084	1382	152.4	72.2	68.4-76.3	554	61.1	26.9	24.7-29.3
Combined	1968-1972	1910627	656	34.3	19.5	18.0-21.1	480	25.1	15	13.6-16.4
Combined	1973-1977	2280123	854	37.5	21.9	20.5-23.5	710	31.1	18.9	17.6-20.4
Combined	1978-1982	2756074	1211	43.9	26.2	24.7-27.8	1070	38.8	23.7	22.3-25.2
Combined	1983-1987	3378400	1696	50.2	30.4	28.9-31.9	1377	40.8	24.9	23.6-26.3

Combined	1988-1992	4024035	2561	63.6	37.9	36.4-39.4	1765	43.9	26.8	25.5-28.1
Combined	1993-1997	4656900	3495	75	42.9	41.4-44.4	2131	45.8	27.9	26.7-29.1
Combined	1998-2002	5284300	5404	102.3	54.9	53.4-56.4	2540	48.1	27.2	26.1-28.3
Combined	2003-2007	5745400	6614	115.1	58	56.6-59.5	2995	52.1	26.7	25.7-27.7
Combined	2008-2012	6340956	8209	129.5	62.2	60.9-63.6	3479	54.9	25	24.2-25.9
Combined	2013-2017	6842573	10362	151.4	69.5	68.1-70.8	4191	61.2	25.1	24.3-25.9
Combined	2018-2022	7290586	12452	170.8	75.2	73.9-76.6	4920	67.5	25.1	24.4-25.8

Table S2. Sensitivity analysis of nested APC model comparisons and dispersion ratios among Indian women using different imputed values (1, 2, 3 and 4) for suppressed counts. Imputation values refer to the values substituted for suppressed registry counts (<5 incident cases). Age-period cells absent from the case extract were treated as zero cases in all scenarios and are distinct from suppressed cells. Models were fitted to the complete 12 × 11 age-period grid, with person-years for every cell. APC analyses were of breast and colorectal cancer incidence in females aged 25 to 84 in Singapore from 1968 to 2022, with 1970 as the reference period and 1890 as the reference cohort.[1]

			Breast				Colorectal cancer			
**Ethnicity**	**Model**	**Effect tested**	*1*	*2*	*3*	*4*	*1*	*2*	*3*	*4*
**Indian**	Age	-	-	-	-	-	-	-	-	-
	Age + Drift	Drift	< 0.001	< 0.001	< 0.001	< 0.001	< 0.01	0.207	0.381	< 0.01
	Age + Cohort	Cohort given drift	< 0.01	< 0.05	< 0.05	< 0.05	0.806	0.264	0.051	< 0.05
	Age + Period + Cohort	Period given cohort	0.543	0.558	0.404	0.207	0.684	0.096	< 0.05	< 0.01
	Age + Period	Cohort given period	< 0.05	< 0.05	< 0.05	< 0.05	0.915	0.706	0.452	0.257
	Dispersion ratio (χ^2/df)	-	1.206	1.140	1.220	1.369	1.425	1.101	1.052	1.107

1 Values are Benjamini–Hochberg-adjusted p-values from quasi-likelihood F-tests of nested APC models. The deviance difference per degree of freedom was divided by a dispersion factor, calculated as the Pearson chi-squared divided by the residual degrees of freedom of the full APC model, with a minimum value of 1. The corresponding dispersion ratios (χ^2/df), before applying this minimum, are presented in the final row. P-values were obtained from the F distribution, using the test degrees of freedom and the residual degrees of freedom of the full model, and were adjusted for multiple testing separately for each imputation value across all model comparisons for both cancers and all ethnic groups (Chinese, Indian, Malay and combined). The comparisons adjusted together also include the test of period given drift, which is not displayed, and only Indian women are shown. Age + Drift is compared with Age, Age + Cohort with Age + Drift, and Age + Period + Cohort with Age + Cohort, so a p-value below 0.05 for Age + Period + Cohort indicates that adding period to the Age + Cohort model significantly improved fit. Age + Period is compared with the full age-period-cohort model, which tests the cohort effect given period. These p-values differ from the unadjusted Poisson likelihood-ratio p-values reported in Sim et al. [22].
